# Under pressure: Effect of a ransomware and a screen failure on trust and driving performance in an automated car simulation

**DOI:** 10.3389/fpsyg.2023.1078723

**Published:** 2023-03-01

**Authors:** William Payre, Jaume Perelló-March, Stewart Birrell

**Affiliations:** National Transport Design Centre, Centre for Future Transport and Cities, Coventry University, Coventry, United Kingdom

**Keywords:** automation, driving, trust, safety, ransomware, failure, performance, cybersecurity

## Abstract

One major challenge for automated cars is to not only be safe, but also secure. Indeed, connected vehicles are vulnerable to cyberattacks, which may jeopardize individuals’ trust in these vehicles and their safety. In a driving simulator experiment, 38 participants were exposed to two screen failures: *silent* (i.e., no turn signals on the in-vehicle screen and instrument cluster) and *explicit* (i.e., ransomware attack), both while performing a non-driving related task (NDRT) in a conditionally automated vehicle. Results showed that objective trust decreased after experiencing the failures. Drivers took over control of the vehicle and stopped their NDRT more often after the explicit failure than after the silent failure. Lateral control of the vehicle was compromised when taking over control after both failures compared to automated driving performance. However, longitudinal control proved to be smoother in terms of speed homogeneity compared to automated driving performance. These findings suggest that connectivity failures negatively affect trust in automation and manual driving performance after taking over control. This research posits the question of the importance of connectivity in the realm of trust in automation. Finally, we argue that engagement in a NDRT while riding in automated mode is an indicator of trust in the system and could be used as a surrogate measure for trust.

## 1. Introduction

Nowadays, connectivity, software, and data storing vulnerabilities are challenges yet to be overcome in the realm of cybersecurity and computers (Seetharaman et al., 2020; ISO/SAE 21434, 2021). These challenges include both automated and connected driving systems, which are not immune to software and hardware failures. Indeed, because they are connected to wireless networks, modern vehicles become more accessible and vulnerable to wrongdoers (Sheehan et al., 2019). Furthermore, more vehicular connectivity results in increasing user content value with respect to private and personal information (Deng et al., 2020) making connected and automated vehicles (CAV) worthy targets for cybercriminals. From a psychology perspective, these issues raise questions on how users apprehend such vulnerabilities and how they react when exposed to cyberattacks while their car is in automated driving mode. Previous research showed drivers had concerns about CAV vulnerabilities (Schoettle and Sivak, 2014; Bansal et al., 2016), with implications for data security, privacy (Payre and Diels, 2020), and safety (Lee and Hess, 2022). The academia and legislators back up drivers’ concerns, especially regarding road safety (ISO/TR 4804, 2020; Dede et al., 2021), stressing that cybersecurity and road safety are linked (Trope and Smedinghoff, 2018). A recent example is a recall issued by the National Highway Traffic Safety Administration (NHTSA; Ridella, 2021) for Tesla vehicles: the turn signals sometimes failed to activate in automated mode due to in-vehicle screen failures. The name of this type of malfunction is silent failure because users are not notified about it. In addition, there is sparse scientific literature on the effect of a cyber-intrusion within the vehicle system, for instance a ransomware attack, on drivers’ behavior and attitudes. Previous research showed that, with respect to declarative data, cyberattacks negatively affected trust (Payre et al., 2022). In fact, trust, as “the attitude that an agent will help achieve an individual’s goals in a situation characterized by uncertainty and vulnerability” (Lee and See, 2004), can drive users’ behavior and engagement with automated vehicles (Perello-March et al., 2023). Yet, little is known on the effect of these realistic use-cases of screen failures on road safety, driver’s attitudes and behavior. Hence, the present study aims to bridge that gap in the literature. The research question of this piece of research is “how cyberattacks and screen failures affect drivers” trust and performance in connected and automated vehicles?’ The focus of this experiment is *whether* and *how* participants resume control when exposed to silent or explicit failures while riding in an automated car and engaged in a non-driving related task (NDRT). The specificity of this study is that there are no takeover requests: drivers are neither encouraged nor asked to resume manual control of the vehicle, but takeover is possible if requested by the driver at any time during the scenario. Resuming control in such instances could be an objective measure of distrust (Lee and See, 2004), with drivers selecting to take over control rather than let the vehicle being driven by an automated and connected system. Similarly, engagement in a NDRT after a system failure, without a takeover request, may demonstrate to what extent drivers trust the automated driving system – note that trust and distrust are two distinct yet related constructs (Lewicki et al., 1998). To this day, previous research does not seem to have investigated this question, hence the present study aims to close this gap in the literature by allowing the driver to select whether they take control of the vehicle following a silent or explicit failure. Driving performance will be measured with respect to lateral and longitudinal control of the vehicle. In addition, the effect of both types of failures on drivers’ subjective trust and attitudes is investigated to further the understanding of drivers’ concerns over CAV vulnerabilities. We hypothesize that the explicit failure (i.e., ransomware) will have a more negative effect on both trust (H1) and manual driving performance (H2) than the silent failure (i.e., no turn signals). Driving performance is expected to be compromised after a manual takeover because drivers need to control a vehicle in a dynamic and demanding environment.

## 2. Materials and methods

### 2.1. Driving simulator

**Figure 1:**
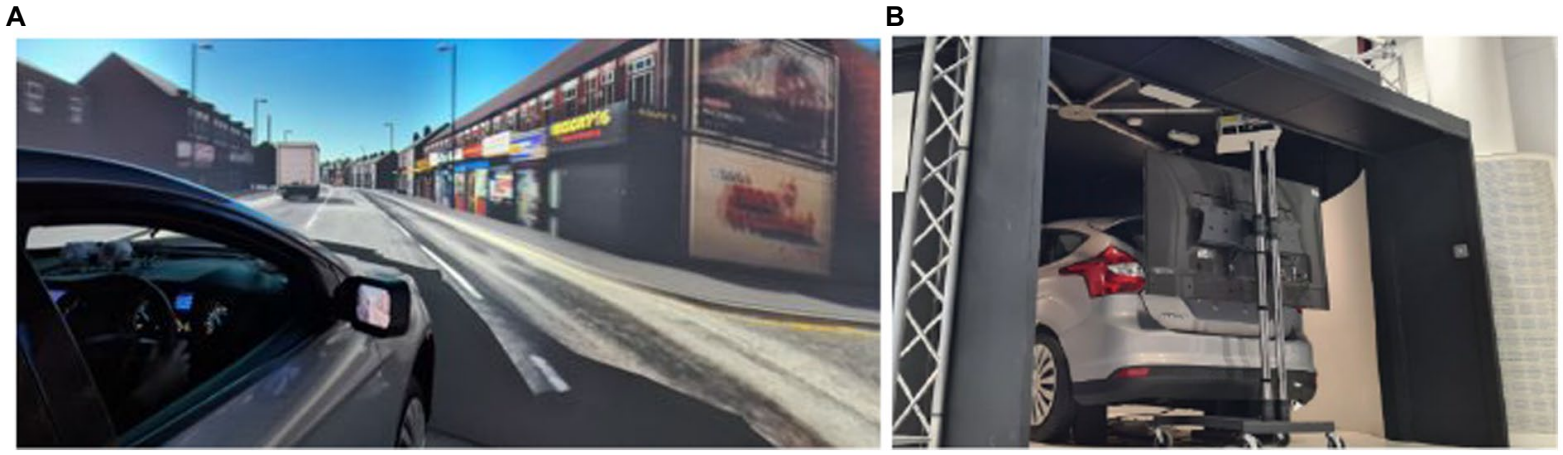
**(A)** (left) Snapshot of the driving simulator and the virtual environment projected on a curved screen. **(B)** (right) View of the back of the car, where a monitor displayed the rear-view mirror.

**Figure 2:**
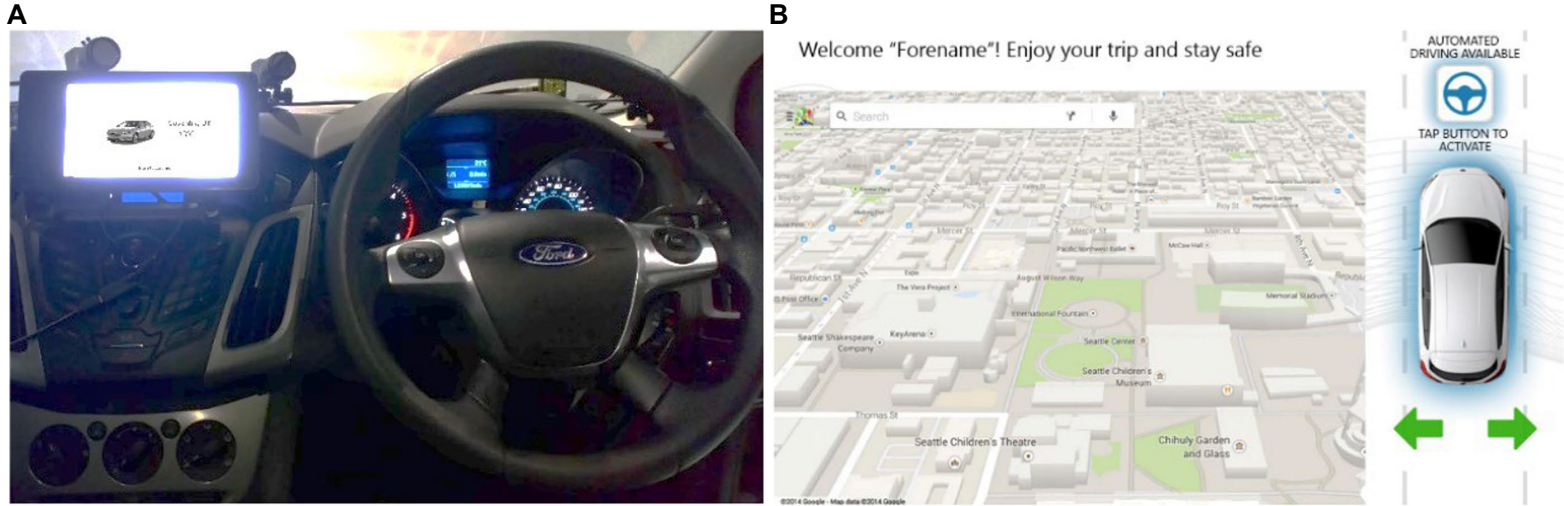
**(A)** (left) The touchscreen fitted on the Ford Focus infotainment system and **(B)** (right), the visual displayed on the touchscreen.

The trials took place in a high-fidelity driving simulator equipped with a moving base, a full-body Ford Focus and realistic graphics generated with Unity (Figures 1A,B). Five projectors provided the visuals with a 1,920 × 1,200 px display resolution at 60 Hz, rendered on a 4.75 m × 2 m, 270° curved screen. A hydraulic system generated road motion with three degrees of freedom. The road environment sound was played in stereo *via* 2 × 20 W speakers. The automated driving simulation included the following features: adaptive cruise control, emergency braking, lane-keeping, and overtaking maneuvers. Uniquely for this study, the driver-in-the-loop simulator allowed the driver to engage the automated driving mode and resume manual control at any time. Automated mode was activated by tapping a blue steering wheel icon on the in-vehicle interface (Figure 2B). Drivers could resume control by either tapping the same icon, using the steering wheel or pressing one of the pedals. The freedom of driving mode (manual or automated) given to the drivers allowed for a more naturalistic experimental environment.

### 2.2. Human–machine interface

A 7″ resistive touchscreen display ran by a Raspberry Pi 3 was fitted on the central console. It hosted an in-house python infotainment interface that was communicating with the driving simulator (Figure 2A). A static map was displayed on the left side of the touchscreen whereas the status of the vehicle was shown on the right-hand part. The vehicle status consisted of showing the mode of control (i.e., manual or automated) and whether the turn signals were activated (Figure 2B).

### 2.3. Experimental procedure

Before entering the driving simulator, participants completed an informed consent form, answered demographic questions and filled in a questionnaire (see Measures). Participants were informed that they would be testing a conditionally automated vehicle, meaning the vehicle was capable of maintaining longitudinal and lateral control, and overtake slower vehicles. Drivers were also told that, ultimately, they were responsible for the driving task if the system failed. Then, they were invited into the simulator for a familiarization trial and drove for at least 5 min to get used to the controllers and dynamics of the vehicle. They were asked to comply with the UK Highway Code and drive in a natural manner. Thereafter, the experimenter explained what the capabilities of a conditionally automated car (SAE-L3) were and how to activate them in the driving simulator. During the familiarization trial, the HMI prompted participants to activate the automated driving mode by tapping a steering wheel icon on the in-vehicle touchscreen (Figure 2B). The vehicle drove automatically for 2 min on a motorway and then safely stopped after pulling-over in a safe area.

**Figure 3:**
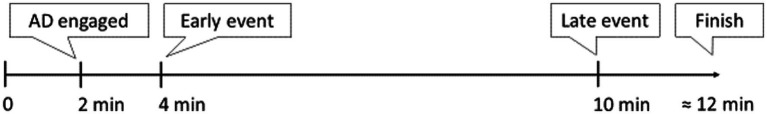
Timeline of the scenario (AD: Automated Driving).

Each participant completed two experimental conditions and one control condition in a counterbalanced order. The events happened either early or late in the scenario to prevent participants from anticipating them. Each condition lasted for 12 min, with participants starting in manual mode in an urban driving environment replicating the city of Coventry, United Kingdom, before merging in a motorway and activating automated driving (Figure 3).

**Figure 4:**
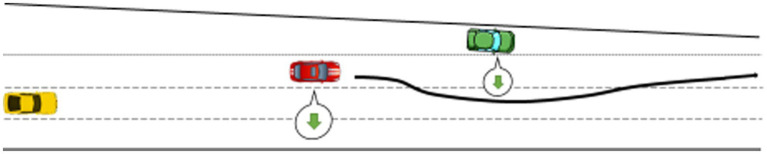
Control condition: the ego vehicle, in red, gives way to the green vehicle merging-in and then overtakes it.

**Figure 5:**
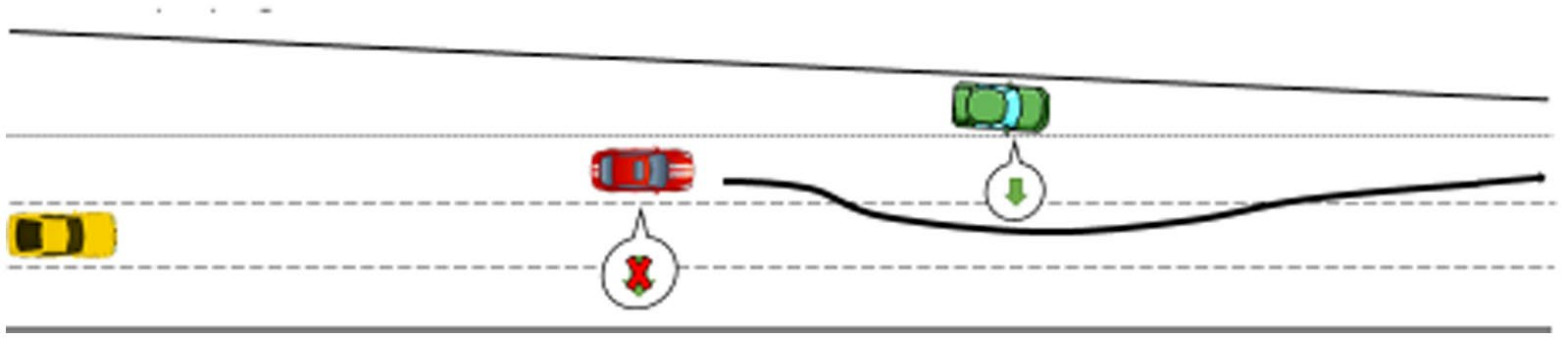
Silent failure condition: the ego vehicle gives way to the green vehicle merging-in and then overtakes it despite the turn signals fail to activate.

**Figure 6:**
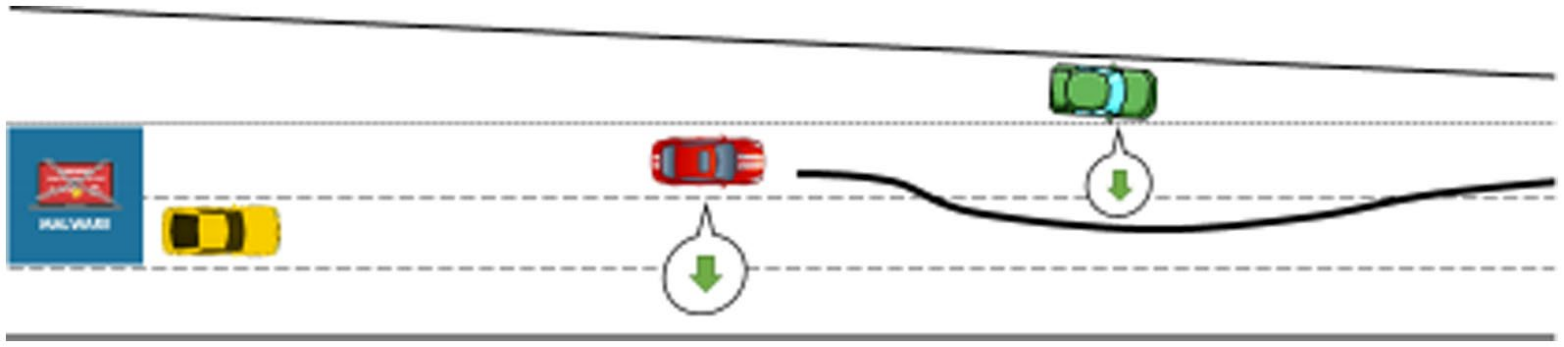
Explicit failure condition: the ego vehicle gives way to the green vehicle merging-in and then overtakes. A ransomware appears on the touchscreen when automated vehicle initiates the overtaking maneuver.

Participants drove on the left-hand side of the road, as required in the UK. They were asked to perform a non-driving related task (NDRT) after activating automated driving. The NDRT was a pen and paper word search grid on a clipboard, which is a visually demanding task inciting drivers to look away from the road. They were asked to circle as many words as possible during each condition. The experimenter explicitly allowed participants to take over control and reengage automated driving whenever they wanted. A 5-min break between each condition was implemented, during which a series of questions on the experimental condition the driver had just completed were administered. The simulated driving scenario included two similar overtaking maneuvers per trial: one successful, the other leading to either a silent or an explicit failure. The first event happened after 4 min and the second one after 10 min (Figures 4–6).

**Figure 7:**
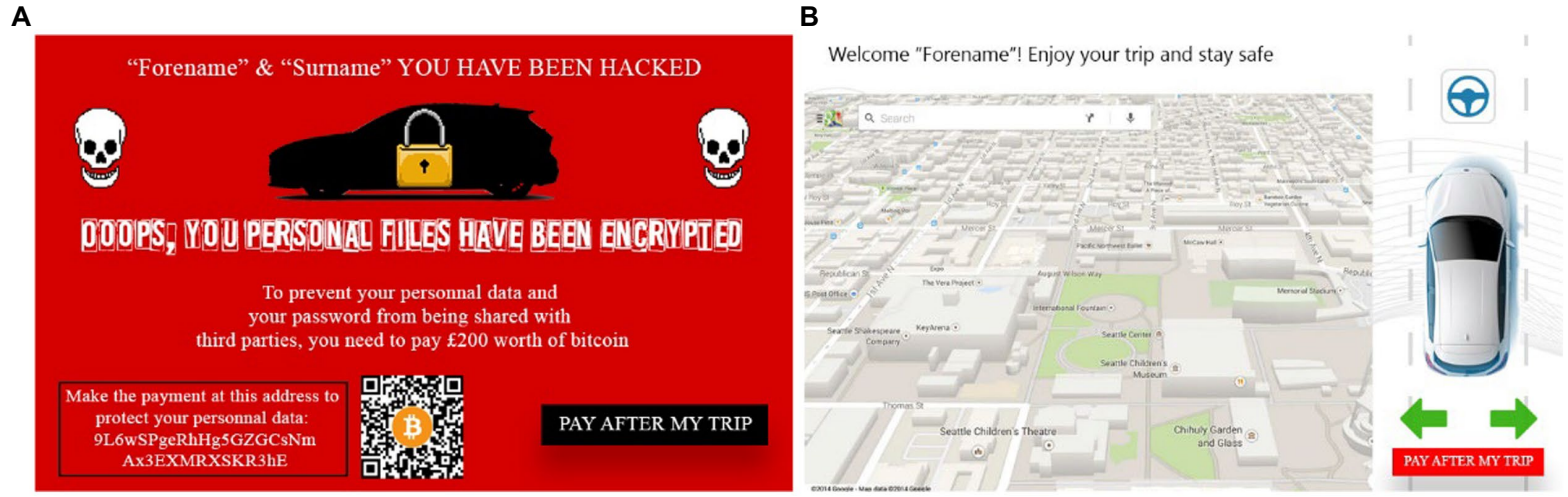
**(A)** (left) The ransomware as it appeared on the touchscreen, **(B)** shows the HMI after tapping the “Pay after my trip” on the bottom right corner.

In the explicit condition, a cyber-attack was simulated. As soon as the automated vehicle started one of the two overtaking maneuvers per condition, a ransomware popped on the in-vehicle touchscreen. The visual was inspired by both the Wannacry ransomware from 2017 and Wolf and Lambert (2017). The name of the participants appeared on top of the screen, along with a message demanding them to pay £200 worth of Bitcoin to protect their personal data that had been encrypted by the ransomware. Indeed, at the beginning of the experiment, participants entered personal information on the touchscreen (name, surname, email, and password) to personalize the messages they would see during the trials and increase their level of involvement when exposed to the ransomware. None of this information was stored. Participants could tap the “Pay after my trip” button to go back to the GPS and automation status screen (Figures 7A,B). The performance of the automated driving system was not altered by any means during the explicit condition, whether participants tapped the button or not.

### 2.4. Participants

A convenience sample of 38 adult volunteers was recruited, although only 37 completed the 2 h-long study after one of them withdrew due to simulator sickness (15 women and 22 men, *M*_age_ = 36.2, SD = 12.5). Their average yearly mileage was 7,737 miles (min = 0, max = 20,000, SD = 5,891) and their driving experience ranged from 0 to 43 years (*M* = 15.7, SD = 13.1). They were free to withdraw at any time. All of the participants held a valid driving license. They had normal or corrected to normal vision and were at least 18 years old. They were compensated for their time with a £20 voucher. The experiment was reviewed and approved by Coventry University ethics committee.

### 2.5. Measures

#### 2.5.1. Declarative trust and intention to pay the ransom

**Figure 8:**
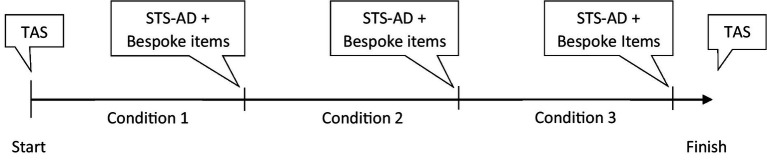
Timeline of the trust scales administration across the whole experiment.

To assess the different layers of trust in automation (i.e., dispositional, situational, and learned) (Hoff and Bashir, 2015), we used the Trust in Automation Scale (TAS; Körber, 2018) to measure dispositional trust. The TAS consists of 19 items distributed in six dimensions (i.e., Reliability/Competence, Understanding/Predictability, Familiarity, Intention of Developers, Propensity to Trust and Trust in Automation). In addition, we used the Situational Trust Scale – Automated Driving (STS-AD, Holthausen, 2020) to measure situational trust. The STS-AD is a six-item single factor scale. Additionally, two bespoke items were administered to evaluate learned trust (i.e., *I would recommend someone else to trust this conditionally automated vehicle*, and *I think it is necessary to trust vulnerable conditionally automated vehicles*). In addition, another question on whether drivers had considered paying the ransom (i.e., and the amount in £) was asked (5-point Likert scale ranging from 1: *strongly disagree* to 5: *strongly agree*). See Figure 8 for more details.

#### 2.5.2. Driving behavior and trust

This study adopted a conservative approach and both lateral and longitudinal control were measured for 60 s (see McDonald et al., 2012) after each failure to capture the manifold driving behaviors observed after the failures, as there were no takeover requests, such as noticing the failure, monitoring the road, monitoring the HMI, resuming control of the vehicle and resuming the NDRT. Manual driving performance was measured based on the time spent driving manually within these 60 s. Automated driving performance after each failure were scripted and similar across the control and experimental conditions.

Three measures of behavioral trust (i.e., reliance on the connected and automated vehicle) were collected:

- Taking over manual control: whether participants resumed control after the event (i.e., no failure, silent failure or explicit failure)
- Time driving manually after each failure, measured for 60 s [as opposed to time driving in automated mode in Azevedo-Sa et al. (2021)].
- Resuming the non-driving related task (NDRT): whether participants stopped then resumed the NDRT (i.e., word search) for 60 s after the failure events. Resuming the NDRT could arguably be an indicator of trust as participants decide to not monitor the system or resume manual control of the vehicle.

The following measures related to driving performance and safety were collected:

- Speed homogeneity, a measure of longitudinal control, is the standard deviation of the average speed (van Nes et al., 2010). Lower values indicate that individuals drive at a more consistent speed.
- Mean speed, in miles per hour.
- Standard deviation of the steering wheel angle: lateral control (Lenneman and Backs, 2009).
- Crash (discrete variable).

#### 2.5.3. Data analysis

Statistical analyses were performed using SPSS v.26. The significance level was set at α = 0.05. Self-reported data from the STS-AD, TAS and the two bespoke trust items were analyzed using a repeated measures ANOVA to test for variations in trust across conditions. In addition, because we found a different number of drivers resuming control after each failure, we considered splitting our participants into two groups for data analysis, depending on whether they had resumed control or not after each failure. Hence, we conducted one-way ANOVAs to test for the effect of whether drivers resumed control after a failure on situational trust. The TAS and the two bespoke items did not meet the normality assumption so we conducted independent-samples Mann–Whitney U tests instead, followed by Wilcoxon tests for pair-wise comparisons.

Regarding behavioral measures of trust, pair-wise *t*-tests were conducted to test for the effect of the failures on taking over manual control, time driving manually and resuming the NDRT.

Ultimately, for driving performance measures, the data was not normally distributed for the standard deviation of the steering wheel angle, speed homogeneity and mean speed. Following the same criterion used for the self-reported data of splitting participants in two groups depending on whether they had resumed control or not after the failures, we conducted independent-samples Mann–Whitney *U*-tests for between-subjects comparisons and Wilcoxon tests for pair-wise comparisons.

## 3. Results

Observations were missing for five participants in the silent condition and four in the explicit due to the simulation software failing to record data.

### 3.1. Questionnaires, participants’ characteristics, and intentions

**Figure 9:**
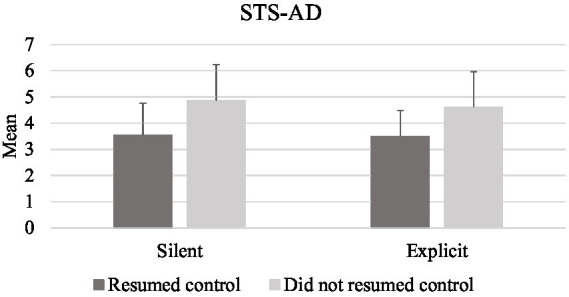
Mean scores for the STS-AD scales in both experimental conditions.

Trust scores did not differ significantly between conditions for the Situational Trust Scale – Automated Driving (STS-AD) (*F* (2, 68) = 0.883, *p* = 0.418). Similarly, Trust in Automation Scale (TAS) scores did not differ before and after the study. Further analyses were conducted to understand the links between declarative trust and driving behavior, more specifically resuming control after the failures. With respect to the STS-AD, a one-way ANOVA showed that trust scores were higher for those who did not resume control after both the silent (*F*(1, 34) = 4.67, *p* = 0.038) and explicit failures (*F*(1, 36) = 5.09, p = 0.03) compared to those who did (Figure 9).

**Figure 10:**
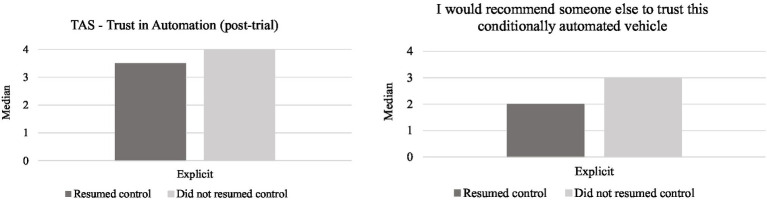
Median values for the trust in automation dimensions and bespoke items depending on whether participants resumed control after the explicit failure.

The dimensions of the TAS and the bespoke items did not meet the normality assumption so independent-samples Mann–Whitney U tested for the effect of resuming control during each type of failure on trust. Results did not show differences between the pre and post scores of the TAS scale. However, further analysis results revealed that the post-trial scores of the TAS dimension of *trust in automation* (*U* = 62.5, *p* = 0.023) were higher for drivers who did not resume control after the explicit failure (Mdn = 4, IQR = 1.5), compared to those who did (Figure 9). Regarding the bespoke item on recommending trust in an automated vehicle, drivers who did not resume control in the explicit condition scored higher (Mdn = 3, IQR = 2) than those who did (*U* = 47.5, *p* = 0.004; Figure 9). No significant differences were found for the bespoke items on trust in the silent condition (see Figure 10).

Additional analyses were conducted to explore whether sex and age (i.e., median split, Mdn = 33) had an effect on declarative trust, but no significant effects were found. However, younger participants (i.e., <33 years; *M* = 2.53, SD = 1.93) indicated they had a stronger intention to pay the requested £200 to protect their personal data than older drivers (*M* = 1.11, SD = 0.32; *F*(36) = 9.46, *p* = 0.004; η^2^ = 0.21).

### 3.2. Trust and driving behavior

No drivers resumed manual control of the car in the control condition. There were no significant differences between the number of times drivers resumed control after the silent (*n* = 6) and explicit (*n* = 9) failures, however, it may be worth mentioning this trend represented roughly a 1/3 more drivers resuming control after the explicit failure. Regarding the time driving manually after each failure, results showed that there were no significant differences between experimental conditions. With respect to the NDRT, significantly fewer drivers resumed the word search after interrupting it in the explicit condition (*n* = 24), compared to the silent condition (*n* = 32; *t*(34) = 2.50, *p* = 0.017).

### 3.3. Driving performance and safety

#### 3.3.1. Silent failure: No turn signals

The descriptive statistics of the driving performance data are presented in Table 1. Data from five participants was lost due to software issues. There was a significant effect of resuming control on speed homogeneity (*U* = 14, *p* = 0.001) between those who resumed control (Mdn = 1.35, IQR = 2.55) and those who did not (Mdn = 4.66, IQR = 3.36). A similar effect was also observed on the standard deviation of the steering wheel angle (*U =* 32, *p* = 0.025). Those who resumed control showed a greater variation of the steering wheel angle (Mdn = 13.178, IQR = 6.27) than those who did not (Mdn = 7.814, IQR = 8.32).

**Table 1:** Driving performance measures for the silent failure condition. Statistically significant effects are indicated with an asterisk (*).

Resumed control		Mean speed	SD steering wheel angle	Time driving manually	Speed homogeneity
Yes (*N* = 6)	Mean	56.84	13.19	18.72	1.69
	Median	57.53	13.18*	17.01	1.35*
	SD	5.673	3.17	9.55	1.26
No (*N* = 26)	Mean	60.82	8.35	0.00	4.21
	Median	61.12	7.81*	0.00	4.66*
	SD	1.53	4.32	0.00	1.44

#### 3.3.2. Explicit failure: Ransomware

Data from four participants was lost due to data software issues (Table 2). There was a significant difference (*U* = 57, *p* = 0.04) in mean speed between drivers who resumed control (Mdn = 56.59, IQR = 9.86) and those who did not (Mdn = 61.17, IQR = 1.82). A similar effect was also observed on the standard deviation of the steering wheel angle (*U =* 43.5, *p* = 0.007). These values were greater for those who resumed control (Mdn = 13.83, IQR = 5.57) than those who did not (Mdn = 4.32, 9.31).

**Table 2:** Driving performance measures for the explicit failure condition.

Resume control		Mean speed	SD steering wheel angle	Time driving manually	Speed homogeneity
Yes (*N* = 9)	Mean	56.59	14.05	31.66	3.61
	Median	56.59*	13.83*	33.71	3.37
	SD	5.3	5.07	18.97	2.02
No (*N* = 24)	Mean	61.18	8.46	0.00	3.86
	Median	61.17*	4.32*	0.00	3.94
	SD	2.03	5.27	0.00	1.51

#### 3.3.3. Critical accident

One participant crashed the vehicle 4.02 s after being exposed to the ransomware and taking over control.

## 4. Discussion

This research investigated the effect of different system failures (i.e., silent and explicit) on self-reported and behavioral trust in automation during conditionally automated driving. Because resuming control from an automated driving system on a motorway with vehicles driving around is a complicated and hazardous task, we hypothesized that the explicit failure (i.e., ransomware) would have a more negative effect on both trust (H1) and manual driving performance (H2) than the silent failure (i.e., no turn signals).

Results from the subjective and objective measures provided evidence supporting H1. Ratings from all trust scales (i.e., the STS-AD, the TAS and the two bespoke items) were aligned, indicating that drivers who chose to resume control after experiencing a failure had lower levels of trust than when there was no failure. This was prominent after the explicit failure. Automation failures are expected to decrease trust (Lee and Moray, 1992; Parasuraman and Riley, 1997). However, when drivers are engaged in a NDRT, they are more likely to miss silent failures than explicit failures, because the former are not salient (Parasuraman and Riley, 1997). This would support our finding that the explicit failure had a greater negative effect on trust ratings than the silent one. Participants’ intention to pay the ransom was low, which is in line with recommendations from UK’s independent authority on public’s information rights to not pay ransom demands (Information Commissioner’s Office, 2022). Results also indicated that younger drivers were more keen to pay than older ones, suggesting that prevention campaign on cybersecurity should target younger individuals in priority (e.g., 33 years old in this study).

Resuming manual control after experiencing a system failure was a determinant of trust ratings as declarative trust scores dropped after both failures. Surprisingly, the type of system failure had no significant effect on two out three measures of driving behavior related to trust. Time driving manually and the number of manual control takeovers, which was relatively low (i.e., *N* = 6 in the silent condition, *N* = 9 in the explicit), did not significantly differ across experimental conditions. Among the six participants who resumed control after the silent failure, only one declared having noticed the missing turn signals (Payre et al., 2022). One explanation is that the lack of system transparency (i.e., how the system explicitly informs the user about its status and operations) led these six participants to resume control. The reason why only nine drivers resumed control after the explicit failure could be that some participants focused on the ransomware and interacted with the in-vehicle screen rather than taking over control. This suggests that, if the car in automated driving mode operates adequately, drivers try to understand and monitor the situation (as indicated by eye glance behavior; Payre et al., 2023) before making an intervention.

It is likely that resuming manual control was not a response exclusively toward the screen failure in most cases, but an indicator of lack of situational trust due to the driving context. It has been argued that malfunctions *per se* do not have a detrimental effect on trust, unless malfunctions impair drivers’ capability to mitigate the risk of a negative outcome (Seet et al., 2020). Hence, the lack of system transparency and feedback due to screen failures possibly exacerbated distrust during a potentially hazardous driving condition (see Figures 8, 9), leading to a lack of reliance in automation, disuse of automation, and eventually manual takeover. Supporting this statement, Kraus et al. (2020) found that trust decreased after drivers experienced malfunctions with low system transparency in a simulated automated driving study. In the present study, the lack of system transparency – i.e., the system does not tell drivers it is failing, or does not provide sufficient level of information on its status – led some participants resuming control after both failures.

Participants had significantly lower levels of engagement in the NDRT in the explicit condition, after failure, than in both the silent and control conditions. This result could mean that drivers were suspicious after the ransomware and either resumed control, despite the absence of a specific takeover request from the vehicle, or they monitored the environment to ensure the vehicle was driving appropriately. Not resuming the NDRT supported the data showing that the ransomware (explicit failure) had a more detrimental effect on trust than the missing turn signals (silent failure). This is congruent with the results on declared and observed trust (i.e., resuming manual control), and this is a novel contribution from this study: in the absence of a takeover request, drivers’ engagement in the NDRT is an indicator of trust in the system and could be used as a surrogate measure for trust. Unfortunately, we did not collect word search task completion scores after each condition but only after completing the whole experiment. This would have allowed us to correlate NDRT engagement with trust scales, and potentially establish a link between behavioral and declarative trust.

Regarding driving performance data, results indicated that, compared to automated driving, resuming manual control after experiencing a silent or explicit failure decreased performance in some instance. H2 is partially supported. This result is not surprising as resuming manual control of the vehicle at high speed with other vehicles around is a demanding and hazardous situation. Lateral control – i.e., standard deviation of the steering wheel angle (SDSWA) – was significantly impaired when drivers resumed control after both types of failures. Lateral control has been found to be an indicator of impaired driving in previous work (Das et al., 2012; Naujoks et al., 2016). Regarding longitudinal control, speed homogeneity was also affected when drivers resumed control after the failures, but not in the expected way. Indeed, lower values indicate that individuals drive at a more consistent speed (van Nes et al., 2008, 2010). In the present study, speed homogeneity was better in manual than in automated driving after both failures. One explanation for this is that before the failures, the car in automated driving mode initiated a takeover maneuver (see Figures 5, 6), meaning that the car was accelerating. When participants resumed control during that maneuver, it could be that they maintained the speed at which they resumed control to exercise caution. It also demonstrates that, despite the demanding, sudden and hazardous situation, all but one driver who resumed control managed to safely handle longitudinal control of the vehicle. Mean speed decreased consecutively to the ransomware, but this does not necessarily result in safety issues. Actually, this could be attributed to a compensatory behavior similar to that observed when drivers are engaged in phone conversations, they tend to lower the driving speed to compensate for high information load (De Waard, 1996; Rakauskas et al., 2004). This coping mechanism is considered a compensatory behavior and has been related to increases in mental workload (De Waard, 1996) which, in this case, would be associated with the ransomware popping on the in-vehicle screen unexpectedly. In favor of this statement, work in the human-computer interaction domain has found mental workload to increase after computer malfunctions (Hirshfield et al., 2014). Overall, the explicit failure affected more measures of driving performance, probably because the ransomware was more conspicuous than the missing turning signals from the silent failure.

What is specific to the present study is that there were no takeover requests, and failures did not result in degraded automated driving performance. Despite the automated vehicle driving adequately under both the silent and explicit failures, a number of participants decided to resume control, which resulted in some cases in poorer driving performance in terms of lateral control compared to automated driving. Automation failures raise concerns with respect to road safety, so are vulnerable connected vehicles. This is salient in this study with respect to the participant who resumed control after seeing the ransomware and crashed the vehicle a few seconds later.

The implications of this piece of research are manifold. First, failures related to connected vehicle features negatively affected trust in automation. Similar results have been shown with automated vehicle features. This study uniquely shows that this is also the case with connected features. Although failures bore no influence on automated driving performance, some participants thought it did (for further details see Payre et al., 2022). This observation is new for the automotive literature as previous research suggests that connectivity and automation are often considered overlapping concepts, rather than two distinct technologies not necessarily relying on each other to operate. Future research should further investigate the role of connectivity as a dimension of trust in automated driving. Another implication of this study is that connectivity vulnerabilities lead to worse lateral control of the vehicle after drivers have resumed control. Therefore, not only automation reliability is of paramount importance for safety, but so is security of connected and automated vehicles. In the present study, one participant crashed the vehicle after seeing the ransomware and resuming control.

There were limitations to this study. The first failure event (i.e., early in the scenario) took place on straight portion of the motorway whereas the second one (i.e., late in the scenario) happened at the end of long curve merging onto another motorway. While counterbalancing the events prevented priming participants, the slightly different road environment may have influenced driving behavior and performance. Concerning the context of the explicit failure, the ransomware popped on the screen without any action from the driver. In reality, people click on a link or tap a button for such pop-ups to appear. While driving simulation can be immersive, participants may have reacted and behaved differently in real life settings, which is why further research on this topic should be conducted on road. Finally, further data collection is required, with a focus on lateral (e.g., standard deviation of the lane deviation) and longitudinal (e.g., time headway) control of the vehicle to determine exactly how screen failures affect driving performance when resuming manual control from a conditionally automated vehicle.

## 5. Conclusion

The answer to this paper’s research question is that cyberattacks and screen failures do affect drivers’ trust and performance in connected and automated vehicles. The primary result is that participants did not always resume control of the vehicle after these failures. When participants resumed control, lateral control performance was compromised compared to automated performance. This was not the case for longitudinal control, which demonstrated that drivers coped with the situation surprisingly well in that regard. Subjective trust differed depending on whether participants resume control and the type of failure. Objective trust decreased, which was expected as the vehicle showed vulnerabilities to external (i.e., cyberattack) and internal (i.e., screen malfunction) threats. It was down to individual driver choice if they chose to resume control of the vehicle, as there were no takeover requests, which has not been explored extensively within the literature. Engagement in the NDRT supported subjective measures of trust, and could therefore be used as a surrogate measure of trust in future studies. Finally, connectivity and automated features seem to be different for drivers, which may help refining the concepts underlying trust in automated driving. Indeed, the present paper presents evidence that connected and automated vehicle failures are perceived as independent events, with the vast majority of drivers (i.e., 81% after the silent failure and 73% after the ransomware) trusting the automated vehicle to drive itself, despite a connectivity failure occurring. In case of connectivity issue, drivers may not always resume control. In case of automation issue, they will. This finding will prove very important for automotive research going forward: trust in automation is crucial, and so is trust in automated vehicles security.

## Data availability statement

The raw data supporting the conclusions of this article will be made available by the authors, without undue reservation.

## Ethics statement

The studies involving human participants were reviewed and approved by Coventry University Ethics Committee. The patients/participants provided their written informed consent to participate in this study.

## Author contributions

All authors listed have made a substantial, direct, and intellectual contribution to the work and approved it for publication.

## Funding

This work was supported by the UKRI Trustworthy Autonomous Systems Hub (EP/V00784X/1).

## Conflict of interest

The authors declare that the research was conducted in the absence of any commercial or financial relationships that could be construed as a potential conflict of interest.

## Publisher’s note

All claims expressed in this article are solely those of the authors and do not necessarily represent those of their affiliated organizations, or those of the publisher, the editors and the reviewers. Any product that may be evaluated in this article, or claim that may be made by its manufacturer, is not guaranteed or endorsed by the publisher.
